# Polymorphisms of DNA repair genes are associated with colorectal cancer in patients with Lynch syndrome

**DOI:** 10.1002/mgg3.402

**Published:** 2018-04-17

**Authors:** Abram B. Kamiza, Ling‐Ling Hsieh, Reiping Tang, Huei‐Tzu Chien, Chih‐Hsiung Lai, Li‐Ling Chiu, Tsai‐Ping Lo, Kuan‐Yi Hung, Jeng‐Fu You, Wen‐Chang Wang, Chao A. Hsiung, Chih‐Ching Yeh

**Affiliations:** ^1^ School of Public Health College of Public Health Taipei Medical University Taipei Taiwan; ^2^ Department of Public Health College of Medicine Chang Gung University Taoyuan Taiwan; ^3^ Colorectal Section Department of Surgery Chang Gung Memorial Hospital Taoyuan Taiwan; ^4^ School of Medicine Chang Gung University Taoyuan Taiwan; ^5^ Department of Nutrition and Health Sciences Chang Gung University of Science and Technology Taoyuan Taiwan; ^6^ Institute of Population Health Sciences National Health Research Institutes Miaoli Taiwan; ^7^ Ph.D. Program for Translational Medicine College of Medical Science and Technology Taipei Medical University Taipei Taiwan; ^8^ Department of Public Health China Medical University Taichung Taiwan

**Keywords:** colorectal cancer, DNA repair, Lynch syndrome, polymorphisms, Taiwan

## Abstract

**Background:**

DNA repair genes are crucial for maintaining genomic stability by preventing mutagenesis and carcinogenesis. The present retrospective cohort study aimed at investigating whether *MLH1*,*APEX1*,*MUTYH*,*OGG1*,*NUDT1*,*XRCC5, XPA*, and *ERCC2* single nucleotide polymorphisms (SNPs) are associated with colorectal cancer (CRC) in Chinese population with Lynch syndrome.

**Methods:**

From Amsterdam criteria family registry, we identified 270 patients with Lynch syndrome. Hazard ratios (HRs) and 95% confidence intervals (CIs) for the association between DNA repair SNPs and CRC were calculated using a weighted Cox proportional hazard regression model.

**Results:**

Heterozygous variants of rs1799832 in *NUDT1* (HR = 2.97, 95% CI = 1.51–5.83) and rs13181 in *ERCC2* (HR = 2.69, 95% CI = 1.10–6.55) were significantly associated with an increased risk of CRC compared with wild‐type homozygous CC and TT genotypes, respectively. However, the variant CG+GG genotype of *MUTYH* rs3219489 was associated with a decreased risk of CRC (HR = 0.49, 95% CI = 0.26–0.91) compared with the homozygous CC wild‐type counterparts.

**Conclusion:**

Our findings revealed that polymorphisms of DNA repair genes that include *NUDT1*,*ERCC2,* and *MUTYH* are associated with CRC in patients with Lynch syndrome in Chinese population. Further studies with large sample size are needed to confirm our findings.

## 1 INTRODUCTION

Lynch syndrome is a germline mutation in mismatch repair (MMR) genes (Boland, 2005). *MLH1* (OMIM: 120436), *MSH2* (OMIM: 609309), *MSH6* (OMIM: 600678), *PMS2* (OMIM: 600259), and *EPCAM* (OMIM: 185535) germline mutations are responsible for Lynch syndrome (Lynch et al., 2009). More than 80% of mutation carriers have a germline mutation in *MLH1* and *MSH2* and are at a greater risk of colorectal cancer (CRC) than the general population (Barnetson et al., 2006). MMR genes are crucial for maintaining genomic stability by repairing mutations that occur during DNA replication in preparation for cell division (De Jong et al., 2004). Specifically, *MSH2* is responsible for proofreading a newly synthesized DNA strand for mismatch base pairing, while *MLH1* coordinates the activities of other genes to repair the mismatch mutations (Li, 2008). In addition to the MMR, DNA repair genes such as *APEX1* (OMIM: 107748), *MUTYH* (OMIM: 604933), *OGG1* (OMIM: 601982), *NUDT1* (OMIM: 600312), *XRCC5* (OMIM: 194364), *XPA* (OMIM: 611153), and *ERCC2* (OMIM: 126340) play a crucial role in repairing DNA mutations and thus preventing cancer development (Sancar, Lindsey‐Boltz, Unsal‐Kaçmaz, & Linn, 2004).

However, DNA repair genes are polymorphic, and the single nucleotide polymorphism (SNP) of these genes is associated with cancer development (Moreno et al., 2006). Polymorphisms of *MLH1*,* APEX1*,* MUTYH*,* OGG1*,* NUDT1*, and *XRCC5* are associated with sporadic CRC (Kim et al., 2004; Lai et al., 2016; Yang et al., 2009) and other site‐specific cancers (Li et al., 2011; Savina et al., 2016; Smith et al., 2011). However, recent studies have indicated that *XPA* and *ERCC2* SNPs are not associated with sporadic CRC (Chang et al., 2016; He, Deng, & Luo, 2015). The association between DNA repair genes and CRC in germline mutation carriers has rarely been investigated. Only three studies have investigated this association, and the results have been inconsistent (Garre et al., 2011; Reeves et al., 2012; Win et al., 2013). Of these studies, two have reported that DNA repair genes are not associated with CRC (Reeves et al., 2012; Win et al., 2013). By contrast, Garre et al. reported that *OGG1*,* NUDT1*, and *MUTYH* SNPs are associated with CRC risk (Garre et al., 2011). However, Garre et al. included patients with microsatellite stable‐hereditary nonpolyposis colorectal cancer (MSS‐HNPCC). Patients with MSS‐HNPCC have lower risk of CRC and lack evidence of the MMR deficiencies that define this syndrome (Llor et al., 2005).

Since germline mutation carriers have dysfunctional MMR genes and are at an increased risk of CRC, DNA repair genes are crucial for preventing mutations and cancer development. We therefore investigated whether *MLH1* rs1799977, *MLH1* rs1800734, *APEX1* rs1130409, *APEX1* rs1760944, *MUTYH* rs3219489, *OGG1* rs1052133, *NUDT1* rs1799832, *XRCC5* rs828907, *XPA* rs1800975, and *ERCC2* rs13181 were associated with CRC.

## 2 MATERIALS AND METHODS

### 2.1 Ethical compliance

Protocol of this study was performed under the approval of the Institution Review Boards of the Taiwan National Health Research Institutes and Taipei Medical University. All patients provided an informed consent for their data and biospecimens to be used by Taiwan HNPCC consortium.

### 2.2 Patients

Patients suspected of HNPCC were recruited into the Amsterdam criteria family registry using the guidelines adopted from Amsterdam criteria II as previously described (Kamiza et al., 2015, 2016, 2018; Tang et al., 2009). One thousand and fourteen patients and their relatives from 135 Lynch syndrome families were recruited. Family members and relatives were recruited into the Amsterdam criteria family registry via probands.

Probands who fulfilled Amsterdam criteria were screened for germline mutation in *MLH1* and *MSH2*. Genetic analyses were also performed to family members of probands as previously described (Kamiza et al., 2015, 2016, 2018; Tang et al., 2009). Of these probands and family members, 303 were identified as having germline mutation. Approximately, 10.2% (31) of germline mutation carriers were excluded because their DNA polymorphisms results were unavailable. In addition, two germline mutation carriers were also excluded because they had double mutation in *MLH1* and *MSH2*. Eventually, we recruited 270 patients with Lynch syndrome.

### 2.3 Data collection

Nurses were trained to conduct interviews. Clinical data from probands were collected from May 2002 onwards as previously described (Kamiza et al., 2015, 2016, 2018). In addition, patients with Lynch syndrome were interviewed using structured questionnaire, which included demographic factors, dietary factors, lifestyle factors, medical, and family histories of cancer. All patients were followed up biennially from May 2002 to February 2012 for their recent cancer diagnosis statuses. Age at CRC and other site‐specific cancer diagnoses was confirmed using medical reports, pathology reports, cancer registry reports, and death certificates.

### 2.4 Genotyping of DNA repair genes

Genomic DNA from white blood cells of probands and their family members was used for genotyping *MLH1* (NM_000249.3), *APEX1* (NM_001641.3), *MUTYH* (NM_012222.2), *OGG1* (NM_016828.2), *NUDT1* (NM_002452.3), *XRCC5* (NM_021141.3), *XPA* (NM_000380.3), and *ERCC2* (NM_000400.3) (Table 1). Genotyping was performed using Sequenom iPLEX MassARRAY (Sequenom, Inc., San Diego, CA, USA). Matrix‐assisted laser desorption ionization time of flight (MALDI‐TOF) spectroscopy was performed using the Sequenom MassARRAY platform and iPLEX GOLD chemistry as described in our previous studies (Kamiza et al., 2015, 2016, 2018). We added 10 ng of template DNA in polymerase chain reaction (PCR) mixture containing Qiagen HotStarTaq. We conducted primer extensions and shrimp alkaline phosphatase by using guidelines from Sequenom. Primers used for PCR were from Integrated DNA Technologies (OH, USA). Assays were designed using MassARRAY Assay Design, Version 3.1 (Sequenom). We repeated 10% of randomly selected samples for quality control and results showed 100% concordance for all the SNPs.

**Table 1:** Details of DNA repair genes included in this retrospective cohort study in Taiwan

Gene	dbSNP rs #	Chr	GenBank RefSeq	Protein	Location	OMIM #	MAF CHB
*MLH1*	rs1799977	3	NM_000249.3:c.655A>G	p.Ile219Val	Exon 8	120436	G = 0.0194
*MLH1*	rs1800734	3	NM_000249.3:c.–93A>G	–	Promoter	120436	G = 0.4320
*APEX1*	rs1130409	14	NM_001641.3:c.444T>G	p.Asp148Glu	Exon 5	107748	G = 0.4563
*APEX1*	rs1760944	14	NM_001641.3:c.–473T>G	–	Promoter	107748	G = 0.4029
*MUTYH*	rs3219489	1	NM_012222.2:c.1014C>G	p.Gln324His	Exon 12	604933	G = 0.4369
*OGG1*	rs1052133	3	NM_016828.2:c.948+273C>G	p.Ser326Cys	Exon 7	601982	C = 0.4417
*NUDT1*	rs1799832	7	NM_002452.3:c.357C>T	p.Asp119=	Exon 5	600312	T = 0.0874
*XRCC5*	rs828907	2	NM_021141.3:c.–1428G>T	–	Promoter	194364	T = 0.1942
*XPA*	rs1800975	9	NM_000380.3:c.–4A>G	–	5′UTR	611153	A = 0.4634
*ERCC2*	rs13181	19	NM_000400.3:c.2251T>G	p.Lys751Gln	Exon 23	126340	G = 0.1117

Chr, chromosome; RefSeq, reference sequence; OMIM#, OMIM accession numbers; MAF, minor allele frequency; CHB, Chinese Han Beijing; UTR, untranslated region.

### 2.5 Statistical analysis

All the data analysis was conducted using the Statistical Analysis Software (SAS 9.4). Clinicopathological characteristics of patients with Lynch syndrome were described using descriptive statistics. Time at risk begin at birth and end at CRC diagnosis, death, or loss to follow‐up. Patients with Lynch syndrome who did not receive CRC diagnosis were censored at the date of their last known contact or in February 2012.

A Pearson chi‐squared test was used to examine the differences between the expected and observed genotype frequencies and to assess whether each SNP conformed to the Hardy–Weinberg equilibrium (HWE). Because patients with Lynch syndrome were not randomly ascertained, to avoid ascertainment bias, we performed a weighted cohort approach (Antoniou et al., 2005). Hazard ratios (HRs) and 95% confidence intervals (CIs) for the association between DNA repair SNPs and CRC were calculated using a weighted Cox hazard regression model, in which a *p* value of <.05 was considered to be statistically significant. All analyses were two‐sided and adjusted for sex, frequency of colonoscopy, familial clustering, year of birth (<1940, 1940–1949, 1950–1959, 1960–1969, 1970–1979, and >1980), and MMR gene mutated in multivariate model. In addition, we further adjusted for within‐cluster and family correlations as previously described by Williams (2000).

## 3 RESULTS

Median and mean ages at CRC diagnoses were 44.3 and 45.7 years, respectively. A majority of patients 146 (54.1%) with germline mutation in MMR genes were female (Table 2). Approximately, 70% and 30% of patients with Lynch syndrome harbored germline mutation in *MLH1* and *MSH2,* respectively. During the follow‐up period, 129 (47.8%) of the patients with Lynch syndrome were diagnosed as having pathologically confirmed CRC. Of these, 110 (85.3%) were diagnosed as having proximal colon cancer, whereas 19 (14.7%) were diagnosed as having distal rectal cancer.

**Table 2:** Clinicopathological characteristics of patients with Lynch syndrome in Taiwan

Variables	*n *=* *270	%
Age at diagnosis		
Median (IQR)^a^	44.3	37.5–52.3
Mean (SD)	45.7	11.5
Sex, *n* (%)		
Female	146	54.1
Male	124	45.9
MMR gene mutated, *n* (%)		
*MLH1*	190	70.4
*MSH2*	80	29.6
Colorectal cancer, *n* (%)		
No	141	52.2
Yes	129	47.8
Colorectal cancer site, *n* (%)		
Proximal colon	110	85.3
Distal rectal	19	14.7

IQR, interquartile range; SD, standard deviation.

a IQR (25th–75th percentiles).

The genotype frequencies of each DNA repair SNP conformed to the HWE (*p* value >.05). Table 3 presents the association between polymorphisms of DNA repair SNPs and CRC in patients with Lynch syndrome. The heterozygous CT genotype of *NUDT1* rs1799832 was significantly associated with an increased risk of CRC (HR = 2.97, 95% CI = 1.51–5.83) compared with a wild‐type homozygous CC genotype. In addition, the variant TG genotype of *ERCC2* rs13181 was associated with an increased risk of CRC (HR = 2.69, 95% CI = 1.10–6.55) compared with wild‐type homozygous TT genotype. However, the variant CG+GG genotype of *MUTYH* rs3219489 was associated with a decreased risk of CRC (HR = 0.49, 95% CI = 0.26–0.91) compared with the homozygous CC wild‐type counterparts.

**Table 3:** Polymorphisms of DNA repair genes with colorectal cancer risk among patients with Lynch syndrome

DNA repair genes^a^	Total cohort	Person years	CRC cases	Crude HR (95%CI)	*p* value	Adjusted HR (95%CI)^b^	*p* value
*MLH1* rs1799977							
AA	264	11,076	126	1.00		1.00	
AG	6	200	3	**2.54 (1.30–4.95)**	**.006**	2.17 (0.90–5.16)	.081
GG	0	0	0	–	–	–	–
*MLH1* rs1800734							
AA	101	4,432	50	1.00		1.00	
AG	130	5,385	59	1.18 (0.62–2.21)	.614	1.06 (0.59–1.87)	.852
GG	39	1,459	20	1.21 (0.47–3.09)	.693	0.99 (0.39–2.45)	.981
AG + GG	169	6,844	79	1.18 (0.64–2.17)	.594	1.05 (0.60–1.82)	.865
*APEX1* rs1130409							
TT	116	4,949	61	1.00		1.00	
TG	121	5,001	55	1.18 (0.67–2.06)	.335	1.22 (0.74–2.01)	.432
GG	33	1,327	13	0.62 (0.18–2.08)	.437	0.58 (0.14–2.27)	.434
TG + GG	154	6,328	68	1.08 (0.62–1.88)	.785	1.08 (0.64–1.82)	.761
*APEX1* rs1760944							
AA	88	3,644	39	1.00		1.00	
AC	135	5,623	69	0.95 (0.52–1.72)	.855	0.92 (0.51–1.66)	.791
CC	40	1,675	16	0.89 (0.39–2.02)	.780	0.84 (0.35–1.97)	.691
AC + CC	175	7,298	85	0.90 (0.45–1.75)	.748	0.85 (0.43–1.66)	.642
*MUTYH* rs3219489							
CC	79	3,212	35	1.00		1.00	
CG	130	5,482	63	0.81 (0.43–1.52)	.514	0.77 (0.40–1.49)	.441
GG	49	2,045	21	0.44 (0.17–1.14)	.092	0.44 (0.17–1.09)	.078
CG + GG	179	7,527	84	0.69 (0.34–1.36)	.288	**0.49 (0.26–0.91)**	**.024**
*OGG1* rs1052133							
GG	101	4,271	53	1.00		1.00	
GC	119	4,795	50	0.74 (0.34–1.62)	.459	0.95 (0.44–2.03)	.891
CC	50	2,210	26	1.76 (0.94–3.29)	.076	2.18 (0.99–4.79)	.053
GC + CC	169	7,005	76	1.04 (0.53–1.99)	.915	1.29 (0.66–2.51)	.457
*NUDT1* rs1799832							
CC	230	9,595	107	1.00		1.00	
CT	39	1,661	22	**2.97 (1.57–5.60)**	**.001**	**2.97 (1.51–5.83)**	**.001**
TT	1	20	0	–	–	–	–
*XRCC5* rs828907							
GG	163	6,635	78	1.00		1.00	
GT	79	3,371	31	0.69 (0.33–1.42)	.316	0.81 (0.40–1.63)	.559
TT	17	761	11	1.47 (0.74–2.92)	.266	1.83 (0.79–4.22)	.158
GT + TT	96	4,132	41	0.82 (0.44–1.48)	.501	0.96 (0.52–1.75)	.891
*XPA* rs1800975							
GG	80	3,499	46	1.00		1.00	
GA	131	5,406	51	0.64 (0.29–1.39)	.263	0.68 (0.31–1.49)	.341
AA	57	2,286	30	1.02 (0.53–1.92)	.957	1.01 (0.52–1.92)	.982
GA + AA	188	7,692	81	0.77 (0.42–1.40)	.393	0.80 (0.43–1.46)	.471
*ERCC2* rs13181							
TT	245	10,173	116	1.00		1.00	
TG	24	1,063	12	1.29 (0.34–4.75)	.706	**2.69 (1.10–6.55)**	**.029**
GG	0	0	0	–	–	–	–

The bold values mean that the results was statistically significant.

a If total number not equal to 270, then SNP with missing genotype data.

b Adjusted for sex, colonoscopy, date of birth, familial clustering, and specific mutated MMR gene.

The combined effect of having risky genotypes from *MUTYH* rs3219489, *NUDT1* rs1799832*,* and *ERCC2* rs13181 and risk of CRC in patients with Lynch syndrome is shown in Table 4. The HR revealed that patients with Lynch syndrome who harbored at least one risky genotype were significantly associated with an increased risk of CRC (HR = 2.15, 95% CI = 1.23–3.74, for those with one risky genotype and HR = 4.86, 95% CI= 1.69–13.9, for those with two risky genotypes) compared to those with no risky genotype.

**Table 4:** Combined effect of *MUTYH* rs3219489, *NUDT1* rs1799832*,* and *ERCC2* rs13181 polymorphisms and risk of colorectal cancer in patients with Lynch syndrome

Risky genotype^a^	Total cohort	Person years	CRC cases	Adjusted HR (95%CI)^b^	*p* value
0	141	5,980	65	1.00	
1	97	3,843	45	**2.15 (1.23–3.74)**	**.006**
2	19	876	8	**4.86 (1.69–13.9)**	**.003**

The bold values mean that the results was statistically significant.

a Risky genotype were CC, CT, and TG for rs3219489*,* rs1799832, and rs13181 SNPs, respectively.

b Adjusted for sex, colonoscopy, date of birth, familial clustering, and mutated MMR gene.

## 4 DISCUSSION

DNA repair pathway is crucial for preventing mutagenesis and carcinogenesis. *APEX1*,* MUTYH*,* OGG1*,* NUDT1*, and *XRCC5* are members of base excision repair pathway and are crucial for recognizing and repairing oxidative DNA damage as well as mismatch base pairing and single strand breaks (Moreno et al., 2006). Previous studies investigating this association have reported nonsignificant findings (Reeves et al., 2012; Win et al., 2013). However, Reeves et al. suggested that failure to find an association between DNA repair SNPs and CRC does not rule out the involvement of these SNPs in modifying CRC in germline mutation carriers (Reeves et al., 2012). In the present study, *MUTYH*,* NUDT1,* and *ERCC2* SNPs were associated with CRC.

*MUTYH* has a functional role of repairing 8‐hydroxyguanine mismatches that occur as a result of adenine glycosylase activity (Slupska, Luther, Chiang, Yang, & Miller, 1999). A previous study indicated that the homozygous CC genotype of rs3219489 was associated with an increased risk of CRC when compared to the GG genotype (Picelli et al., 2010). In this study, the G allele was protective, which is in line with the findings of Picelli et al. However, a case–control study reported nonsignificant results (Garre et al., 2011). The nonsignificant results reported by Garre et al. may be due to inadequate sample size and different study population as Garre et al. included patients with MSS‐HNPCC. The protective effect observed among those with G allele maybe due to its high efficiency in repairing 8‐hydroxyguanine mismatches that occur during DNA replication in preparation for cell division (Yamane et al., 2003).

The present study has revealed that rs1799832 in *NUDT1* was significantly associated with CRC risk, which is in line with a previous study (Garre et al., 2011). In contrast, a case–control study from Germany suggested that rs1799832 was not associated with oral cancer (Görgens et al., 2007). However, this study included only 29 patients with oral cancer. Moreover, rs1799832 deviated from HWE. NUDT1 also known as MTH1 hydrolyses 8‐oxoguanine‐triphospate (8‐oxo‐dGTP) to 8‐oxoguanine‐monophosphate (8‐oxo‐dGMP), thus preventing incorporation of 8‐oxo‐dGTP into the nascent DNA strand (Nakabeppu, 2001). Rs1799832 is a C to T silent SNP occurring in exon 5 of *NUDT1* (Wu et al., 1995). However, variation in this gene decreases NUDT1 enzyme activity (Maki & Sekiguchi, 1992), hence increasing the risk of CRC among those with a variant genotypes.

Previous studies in Taiwan have suggested that *ERCC2* rs13181 is not associated with CRC (Chang et al., 2016; Yeh, Sung, Tang, Chang‐Chieh, & Hsieh, 2005), which contrasts with our findings. In this study, *ERCC2* rs13181 significantly increased the risk of CRC. The discrepancies observed may be due to different study populations. *XPA* and *ERCC2* are members of the nucleotide excision repair pathway and are involved in repairing and removing DNA adducts (Braithwaite, Wu, & Wang, 1999). *ERCC2* encodes helicase that unwinds the helix region of the damaged DNA to initiate repairing mechanism (Reardon & Sancar, 2002). Variation in *ERCC2* is associated with a low DNA damage repair capacity, which leads to the accumulation of DNA adducts (Spitz et al., 2001), hence increasing the risk of CRC among those carrying the variant G allele. In addition, a recent study also indicated that G allele of rs13181 is associated with CRC (Procopciuc, Osian, & Iancu, 2017), which is in line with our findings. We also assessed the combined effects of having risky genotypes. Our results demonstrated that patients with Lynch syndrome harboring at least one risky genotype in *MUTYH*,* NUDT1,* and *ERCC2* SNPs were at an increased risk of CRC compared to those without risky genotype. Our results support the evidences that CRC is a complex disease caused by complex interactions of different DNA repair pathways (Farrington et al., 2005). However, previous studies reported nonsignificant association between DNA repair genes and CRC (Reeves et al., 2012; Win et al., 2013). The nonsignificant results may be attributed to the differences in ethnicity between Han Chinese and Caucasians. Moreover, Win et al. excluded other types of colorectal polyps, which may have underestimated CRC risk if some of the polyps were malignant.

The main weaknesses of our study are our inability to test *MSH6*,* PMS2*, and *EPCAM*. Almost 54% of the patients were not willing to be followed up, hence, we did not record some newly developed cases. The main strengths of this retrospective cohort study are that all patients include were confirmed to have germline mutation in *MLH1* and *MSH2* and all cancer diagnoses were histologically confirmed.

We have demonstrated for the first time that DNA repair genes are associated with CRC in Chinese population with Lynch syndrome. Our study revealed that *NUDT1* rs1799832 and *ERCC2* rs13181 significantly increased the risk of CRC, whereas *MUTYH* rs3219489 exerted a protective effect.

## CONFLICT OF INTEREST

The authors declare that they have no competing interests.

## AUTHOR CONTRIBUTION

RT, CAH, and CCY conceived and designed the experiments. JFY, WCW, HTC, CHL, LLC, and TPL performed the experiments. ABK, TPL, KYH, CAH, and CCY analyzed the data: CCY, CAH, and RT contributed reagents and analytical tools. ABK, CAH, and CCY drafted the manuscript.
